# Supplementary material for: Investigation on the Potential Application of Na-Attapulgite as an Excipient in Domperidone Sustained-Release Tablets
Source: Molecules. 2022 Nov 26;27(23):8266. doi: 10.3390/molecules27238266 (PMC9738564; doi:10.3390/molecules27238266)
Supplement: Supplementary file 1 [file molecules-27-08266-s001.zip › molecules-2007671-supplementary.pdf]

Investigation on the Potential Application of Na-Attapulgite as an Excipient in Domperidone Sustained-Release Tablets

Yuxuan Xiao [1,2,†], Haiyu Zheng [1,2,†], Meng Du [1,2] and Zhe Zhang [1,2,*]

[1] Key Laboratory of Eco-Environment-Related Polymer Materials, Ministry of Education of China, Lanzhou 730070, China
[2] Key Laboratory of Polymer Materials of Gansu Province, College of Chemistry and Chemical Engineering, Northwest Normal University, Lanzhou 730070, China
* Correspondence: zhangzhe@nwnu.edu.cn; Tel.: +86-13893219765
† These authors contributed equally to this work.

Table S1. Formulation of Domperidone Sustained Release Tablets.

Ingredient (mg)	R1	R2	R3	R4
DOM	10	10	10	10
Na-ATP	5	7.5	10	12.5
Starch	43	40.5	38	35.5
Lactose	20	20	20	20
MCC	20	20	20	20
PVP K30	1	1	1	1
Magnesium stearate	1	1	1	1
Total	100	100	100	100

Table S2. The release data of the drug is applied to the model equation.

Model	Equation
Zero order	$F = k_0 t$
First order	$F = 1 - e^{-k_1 t}$
Higuchi	$F = k_h t^{0.5}$
Korsmeyer-Peppas	$F = k_k t^n$
Weibull	$F = 1 - e^{-t^b/a}$

F, percentage of drug dissolved at time t; k_0, k_1, k_h, k_k, are dissolution rate constants; a is a proportional parameter describing the time dependence; b is the shape parameter describing the dissolution curve.

Table S3. Diameter, hardness, friability and drug content of domperidone sustained release tablets.

Properties	Formulation			
	R1	R2	R3	R4
Diameter (mm)	6.73 ± 0.02	6.74 ± 0.02	6.79 ± 0.04	6.84 ± 0.05
Hardness (kg/cm2)	5.2 ± 0.22	5.0 ± 0.18	4.5 ± 0.11	4.4 ± 0.25
Friability (%)	0.45 ± 0.05	0.52 ± 0.11	0.73 ± 0.08	0.84 ± 0.12
Drug content (%)	98.56 ± 0.55	99.07 ± 0.43	98.89 ± 0.62	98.77 ± 0.91

Table S4. Values of fitted parameters by different drug release models.

Model	Fitted parameters	Formulation			
		R1	R2	R3	R4
Zero order	r2	0.62765	0.5841	0.17767	0.14725
	k_0	0.05309	0.05553	0.03484	0.03326
First order	r2	0.82861	0.88414	0.62856	0.60644
	k_1	0.2299	0.30369	1.00483	1.09587
Higuchi	r2	0.85565	0.82504	0.19106	0.13213
	k_h	0.26574	0.28543	0.30238	0.3019
Korsmeyer-Peppas	r2	0.94517	0.92677	0.99752	0.99914
	k_k	0.40242	0.44271	0.75506	0.77494
	n	0.29225	0.28015	0.02963	0.01453
Weibull	r2	0.96563	0.95802	0.99768	0.99917
	b	0.53094	0.58302	0.07211	0.03554
